# Antifungal mechanism of (*E*)-2-hexenal against *Botrytis cinerea* growth revealed by transcriptome analysis

**DOI:** 10.3389/fmicb.2022.951751

**Published:** 2022-08-22

**Authors:** Ge Song, Shenglong Du, Helong Sun, Quanwu Liang, Haihua Wang, Mingli Yan, Jihong Zhang

**Affiliations:** ^1^Hunan Key Laboratory of Economic Crops Genetic Improvement and Integrated Utilization, Hunan University of Science and Technology, Xiangtan, China; ^2^Department of Chemical Engineering, Xiangtan University, Xiangtan, China

**Keywords:** *Botrytis cinerea*, (*E*)-2-hexenal, RNA sequencing, differentially expressed genes, ergosterol content

## Abstract

Gray mold caused by *Botrytis cinerea*, a necrotrophic plant pathogen, is one of the most damaging diseases of tomato, resulting in both pre- and post-harvest losses. (*E*)-2-Hexenal dose-dependently inhibited the mycelial growth of *B. cinerea*, and caused distortion of mycelia and loss of the cytoplasm content, thus altering the morphology of *B. cinerea* hyphae. To understand molecular processes in response to (*E*)-2-hexenal, transcriptome sequencing was carried out using RNA-Seq technology. RNA-Seq results revealed that a total of 3,893 genes were differentially expressed in *B. cinerea* samples treated with (*E*)-2-hexenal fumigation. Among these genes, 1,949 were upregulated and 1,944 were downregulated. Moreover, further analysis results showed 2,113 unigenes were mapped onto 259 pathways in Kyoto Encyclopedia of Genes and Genomes (KEGG). Moreover, (*E*)-2-hexenal stress affected the expression of genes involved in the pathways of cell wall, cell membrane, and energy metabolism. KEGG pathway analysis showed that the terpenoid backbone biosynthesis and steroid biosynthesis were the most enriched in ergosterol biosynthetic process transcriptome data. Particularly, (*E*)-2-hexenal fumigation had influenced ergosterol biosynthetic gene expression levels (e.g., *ERG1*, *ERG3*, *ERG4*, *ERG7*, *ERG12*, *ERG13*, *ERG24*, *ERG25*, *ERG26*, and *ERG27*), which were in good agreement with the experimental measurement results, and the ergosterol content decreased. Collectively, the results of this study increase our current understanding of (*E*)-2-hexenal inhibition mechanisms in *B. cinerea* and provide relevant information on postharvest shelf life extension and preservation of fruits and vegetables.

## Introduction

*Botrytis cinerea*, a kind of plant necrotrophic pathogen, causes one of the most serious post-harvest diseases that parasitizes on senescent or dead plant tissues and produces gray mold and fruit softening (Blanco-Ulate et al., 2015). Its hyphae can infiltrate plant organs or tissues by means of natural openings or wounds, spread from dead tissues, and then colonize in healthy tissues (Kumar and Kirti, 2015).

It was presumed that a thin or defective cuticle prompted plants to activate defense mechanisms constitutively and secrete antifungal ingredients on their surface, consequently restraining *B. cinerea* development (AbuQamar et al., 2016; Sun et al., 2017). Moreover, cuticle metabolic pathways might immediately regulate interactions of the plant and pathogen *via* intricate lipid signaling pathways (Huysmans et al., 2017) or hormone-regulated defense pathways, resulting in hypersensitive cell death (Mang et al., 2009; Ni et al., 2016).

Ergosterol (ERG) is known as the primary sterol of fungi, except for certain rust fungi, and is of vital importance in cell membranes (Gasecka et al., 2018). Ergosterol takes part in many biological functions, such as regulation, membrane fluidity, cell cycle, and activity (Alcazar-Fuoli and Mellado, 2013; Hokken et al., 2019). About 20 enzymes are involved in the biosynthesis of ergosterol, and its pathway synthesizes squalene from mevalonate gradually (Long et al., 2017). The hydrophobic properties of essential oils (EOs) allows them to easily to enter the lipids of the cell membrane and mitochondrial membrane, thus destabilizing the cell structure, damaging membrane integrity, and increasing cellular membrane permeability (Oliveira et al., 2020). Green leaf volatiles (GLV) are produced in large amounts by green plants, among which (*E*)-2-hexenal is a natural substitute for the antifungal substance with a defensive reaction (Scala et al., 2013), which has a great application prospect in preserving agricultural products (Sun et al., 2022). (*E*)-2-Hexenal, a kind of plant volatile compound, has been well recorded that it can improve plant resistance to pathogens by stimulating accumulation of antifungal substances including PDF1.2, PR-3, and camalexin (Kunishima et al., 2016). It has been proved experimentally that (*E*)-2-hexenal with a concentration of 369 μL/L exerts the highest antifungal effect in all volatile substances (Menniti et al., 2010).

Prost et al. (2005) suggested that trans-2-hexenal has 100% growth inhibition against *B. cinerea* at 100 mM after 24 h of treatment and severely destroys fungal membranes and cell walls. Previous *in vitro* experiments showed that several volatile ingredients in tomato leaves showed inhibitory effects on *B. cinerea* in *Lycopersicon esculentum* (Zhang et al., 2018). Natural plant compounds including citral, benzoic acid, and cuminic acid could hinder respiration enzymatic activity including ATP synthesis and condensation reaction of acetyl-CoA in some fungi (Zheng et al., 2015; Wang et al., 2016). However, the molecular mechanisms of these processes remain unclear and require extensive investigation. Preparation work must be carried out to provide a starting point for in-depth research, particularly into complex mechanisms of (*E*)-2-hexenal on the inhibitory effect of *B. cinerea*. In many biological species, RNA-Seq is used for whole-genome expression analysis and is an increasingly attractive method for reliable and efficient transcriptome sequencing.

Thus far, the molecular mechanism of resistance of (*E*)-2-hexenal to mycelial growth of *B. cinerea* is not fully understood. In this study, the efficacies of (*E*)-2-hexenal in controlling *B. cinerea* infestation and its spore germination were evaluated. To further elucidate the inhibitory mechanism of (*E*)-2-hexenal against *B. cinerea*, changes in global gene expression profiles in *B. cinerea* mycelia between control and (*E*)-2-hexenal treatment were evaluated by transcriptomic analysis, and their impact on the spore germination of *B. cinerea* was analyzed. Physiological and biochemical validation experiments confirmed the transcriptional changes observed. This study provides evidence for identifying genes involved in or some crucial metabolic pathways and developing a safe and effective method for controlling *B. cinerea* infestation in agricultural products using (*E*)-2-hexenal.

## Materials and methods

### Chemicals, fungal culture, and spore suspension preparation

(*E*)-2-Hexenal (purity 99%; CAS: 6728-26-3) was purchased from Sigma-Aldrich (Shanghai, China) and stored in accordance with the manufacturer’s specifications. Propidium iodide (PI) was also acquired from Sigma-Aldrich (Shanghai, China). Isopropanol and trichloromethane were purchased from Sinopharm Group Co., Ltd., (Beijing, China).

*Botrytis cinerea* spores were cultured on potato dextrose agar (PDA) medium (1 L of an infusion from potatoes, 15 g/L agar, and 20 g/L glucose) for 5 days at 28°C. Spores were collected from the *B. cinerea* culture dish with 0.1% (v/v) Tween 80 saline, and then the mycelium was filtered out. The suspension of *B. cinerea* spores was adjusted to 1.0 × 10^6^ spores/mL measured using a hemocytometer.

### Determination of antifungal activity of (*E*)-2-hexenal

Effects of (*E*)-2-hexenal on the mycelial growth of *B. cinerea* were measured *in vitro* by using the agar dilution method (Yahyazadeh et al., 2008). Primarily, high-concentration storage solution (50 μL/mL) of liquid (*E*)-2-hexenal was prepared by dissolving it in anhydrous ethanol and then filtered by filter sterilization. PDA (20 mL) was poured into sterile Petri dishes (90 mm diameter), and the determined amounts of (*E*)-2-hexenal were added to PDA mediums (plus 0.05% Tween 80) to obtain desired concentrations of 0, 10, 20, 40, 80, 160, and 320 μL/L. Inocula with a 6-mm-diameter disk were cut from the PDA plates using a punching bear and then were placed at the center of each new Petri plate. After that, the plates were incubated at 28 + 2°C for 48 h. Each treatment was carried out in triplicate. The percentage of inhibition of mycelial growth (MGI) was computed using the following formula:

MGI(%)=[(dc-dt)/dc]×100

where dc (cm) is the average colony diameter of the control group and dt (cm) is the average colony diameter of the treatment group. The lowest concentration that completely restrained the growth of the fungus was regarded as the minimum inhibitory concentration (MIC). The minimum fungicidal concentration (MFC) was considered as the lowest concentration that restrained the growth of the pathogen in a fresh PDA plate after a 72-h incubation at 28 + 2°C, manifesting more than 99.5% killing of the initial inoculum (Talibi et al., 2012).

### Measurement of extracellular pH and extracellular conductivity

According to the previous method, the extracellular pH and conductivity of *B. cinerea* cells (B05.10), which were obtained from the China General Microbiological Culture Collection Center (Preservation No. CGMCC 3.3790, Beijing, China), were measured using a Delta 320 pH Meter (Mettler-Toledo, Greifensee, Switzerland) and a DDS-12DW Conductivity Meter (Bante Instrument Co., Ltd. Shanghai, China) in accordance with the specifications, respectively (Shao et al., 2013). After the addition of (*E*)-2-hexenal, the extracellular pH and conductivity of *B. cinerea* cells treated at a minimal inhibitory concentration (MIC) or minimal fungicidal concentration (MFC) concentration were selected at 0, 30, 60, and 120 min for treatment. The control did not have any (*E*)-2-hexenal. Results were presented as pH in the growth medium and amount of extracellular conductivity (μs/cm) at each interval of incubation.

### DNA leakage determination

According to the previous method, cell constituents were released into the supernatants and determined with minor modifications (Paul et al., 2011). In brief, *B. cinerea* cells were centrifuged at 4,000 *g* for 20 min and collected from 100 mL potato dextrose broth (PDB), and the particles were eluted three times with sterile water, which were resuspended in phosphate-buffered solution (100 mL, pH 7.0). The suspensions were treated with various concentrations (0, 1 × MIC, and 1 × MFC) of (*E*)-2-hexenal for 0, 30, 60, and 120 min. Subsequently, test specimens (2 mL) were collected and centrifuged for 2 min at 12,000 *g*. In order to measure the concentration of released components, the absorbance of these supernatant solutions (1 mL) was determine at 260 nm by using the UV-2450 UV/Vis Spectrophotometer (SHIMADZU international trade Co. Ltd., Shanghai, China).

### Potassium ions efflux

The amount of the potassium ions was determined according to the method described previously (Tao et al., 2014). After fluid solutions containing *B. cinerea* were exposed to (*E*)-2-hexenal at 1 × MIC or 1 × MFC for a short-term period (0, 30, 60, and 120 min), free potassium ions in suspensions of fungal cells were measured. At each preset interval, the concentration of extracellular potassium was determined by flame atomic absorption spectroscopy following a photometric procedure (Shimadzu AA6300, Japan). Moreover, control in flasks with no (*E*)-2-hexenal was measured. The final results were presented as micrograms per milliliter free potassium ions (μg/mL) in the culture medium in each incubation interval.

### Scanning electron microscopy

The fungi (6-day-old) cultured on potato dextrose agar (PDA) plates were handled with (*E*)-2-hexenal at three concentrations (0, 1 × MIC, 1 × MFC, 8 × MFC, and 16 × MFC) for 2 h and were observed by utilizing scanning electron microscopy (SEM) (Helal et al., 2007; Yahyazadeh et al., 2008). The segments (5 × 10 mm) were removed from PDA plates, quickly placed into 0.05 M phosphate buffer (pH 6.8) containing 3% (v/v) glutaraldehyde, and then stored at 4°C. The samples were stored in this solution for 48 h to fix and then washed with distilled water for three times and 20 min each. Next, they were dehydrated in a series of ethanol solutions (30, 50, 70, and 95%, v/v) for 20 min, respectively, and finally handled with pure ethanol for 45 min. The samples were then dried at the critical point of liquid carbon dioxide. The segments of *B. cinerea* were dried in desiccators until further use. After drying, the prepared samples were placed on 1/2 in SEM stubs with double-slide adhesive labels, and gold–palladium particles were sprayed by a Polaron SEM Coating System sputter coater. The test specimens were observed under a JSM-6360 LV SEM (JEOL, Ltd, Tokyo, Japan), which was operated at 25 kV at 3000 × magnification. All the tests were performed in triplicate.

### Plasma membrane integrity of *Botrytis cinerea* spore assay

Membrane integrity assay was carried out according to the procedure described by Shao et al. (2015). *B. cinerea* spores (5 × 10^5^ spores/mL) were handled with different dosages of (*E*)-2-hexenal (0, 8 × MFC, 16 × MFC). After 2 h of incubation on a rotary shaker which was operated at 25°C at 200 rpm, spores were centrifuged and collected at 8,000 × *g*. Finally, they were stained for 5 min at 30°C with 10 μg/mL propidium iodide (Sigma-Aldrich, Saint Louis, MO, United States) (Qin et al., 2011; Shao et al., 2015). *B. cinerea* spores were then centrifuged and collected, sodium phosphate buffer (pH 7.0) was used, and the spores were eluted twice to remove residual dye. The stained spores were viewed under an Axioskop 40 fluorescence microscope (Zeiss, Oberkochen, Germany) with a separate fluorescein filter set (excitation BP 546/12 nm, emission LP 590 nm, Zeiss no. 15). A total of three different fields from each slide which were no less than 20 spores were randomly chosen, and the spore number in the bright field was regarded as the total spore number.

### RNA sequencing and transcriptomic data processing

Transcriptomic analyses were performed using (*E*)-2-hexenal-treated and untreated *B. cinerea* samples. Hyphae were incubated with 1 × MFC of (*E*)-2-hexenal for 4 h, collected, and frozen in liquid nitrogen for RNA extraction. In brief, total RNA was extracted from control and (*E*)-2-hexenal-treated groups using TRIzol^®^ reagent (Thermo Fisher Scientific Co., Ltd., Shanghai, China) according to the manufacturer’s instructions. RNA integrity was assessed using an RNA Nano 6000 Assay Kit of the Bioanalyzer 2100 system (Agilent Technologies, Palo Alto, CA, United States). Total RNA, 3 μg per sample, was applied as the initial material for RNA sample preparation. The high-quality RNA samples were subsequently used to construct cDNA libraries for sequencing on an Illumina HiSeq platform (Genepioneer Biotechnology Company, Nanjing, China). The raw reads were first processed by a Hiseq2000 system and filtered to eliminate low-quality reads to gain clean reads. The nucleotide genome sequence of *B. cinerea* had been deposited at GenBank, with accession numbers CP009805.1- CP009822.1. The Supplementary Files and transcriptome information had been deposited at the GenBank Sequence Read Archive (SRA) database, with accession number SRR17272254.

### Differential expression analysis and functional enrichment

In order to distinguish these DEGs between the two diverse samples, the expression levels of each transcript were computed through the fragments per kilobase per million mapped fragments (FPKM) method. EdgeR was applied for the differential expression analysis (Lun et al., 2016). The DEGs were chosen according to the following criteria: | log_2_ FC| ≥ 1, the false discovery rate (FDR) less than 0.05, and the FC (fold change) calculated by separating the expression level (FPKM) of each gene in SE1 by that in SE0.

To annotate the function of these differentially expressed genes (DEGs), Gene Ontology (GO) analysis was conducted by using the GOseq R software package (Young et al., 2010). They contained three main categories: cellular component, biological process, and molecular function. The identified DEGs were used as biological pathways enriched through KEGG pathway analyses which were performed by KOBAS software (Mao et al., 2005).

### Real-time fluorescence quantitative PCR

After exposure to (*E*)-2-hexenal at 1 × MFC for 4 h, RNA was extracted from *B. cinerea* cells with TRIzol reagent (Invitrogen, Carlsbad, CA, United States) in accordance with the manufacturer’s instructions. cDNA was synthesized from 2 μg of DNA-free RNA using Moloney Murine Leukemia Virus (M-MLV) Synthesis Kit (Promega, Madison, WI, United States) with oligo dT18. Real-time fluorescence quantitative PCR (RT-PCR) was conducted using a FastStart Universal SYBR Green Master (Roche, Switzerland) on a BIO-RAD CFX Connect real-time PCR detection system. Primers for ergosterol biosynthetic gene which were designed were performed by using Primer Premier for Windows, version 5.0 (Premier Biosoft International, Palo Alto, CA, United States). All primer pairs for expression assays are listed in Table 1. The reaction procedure of RT-PCR was as follows: 95°C for 10 min, followed by 40 cycles of 95°C for 15 s and 60°C for 1 min. The relative expression level was calculated based on the 2^–ΔΔCT^ method using actin gene as an internal reference (Livak and Schmittgen, 2001). In total, three biological replicates were carried out for all qPCR experiments.

**TABLE 1:** Primers used in qRT-PCR analysis.

Genes	Locus	Forward primers (5′→ 3′)	Forward primers (5′ → 3′)
*ERG1* (*squalene epoxidase*)	BCIN_01g00360	TTTTCTCCCTCCATCCACGC	ACGATAACCACACACGCCTT
*ERG3* (*C-5 sterol desaturases*)	BCIN_16g04720	AGGACCGGGTATGTGGTACA	TCTCCTTCCGCCTCCAAAAC
*ERG7* (*2,3-oxidosqualene-lanosterol cyclase*)	BCIN_02g00670	CGGAATGGCCTCAGACAACT	GCGCATGTGAATCCACCATC
*ERG12* (*mevalonic kinase*)	BCIN_05g03770	TCGATGATCTGCCATGGAGC	CAACCTCGTGAGCTGTGGAT
*ERG13* (*3-hydroxy-3-methylglutaryl-coenzyme A synthase*)	BCIN_02g00670	CGGAATGGCCTCAGACAACT	GCGCATGTGAATCCACCATC
*ERG25* (*C-4 sterol methyl oxidase*)	BCIN_01g00350	ACCATTGCTGCAACTCCAGA	ACCGAGACCGAGGACCATAA
*ERG26* (*C-3 sterol dehydrogenase*)	BCIN_16g04070	GTTGATGATTGGGCGCATCC	TTTCGCTTTTTCGCCACTCG
*ERG27* (*3-keto reductase*)	BCIN_03g05110	CCAAACCCTCCGACGATGAA	AATGCTACCCAGACTGGTGC
*Bcrpl5 (Actin)*	BCIN_14g04230	CGTCACTACCTTCAACTCCATC	CGGAGATACCTGGGTACATAGT

### Determination of ergosterol content in cell membranes

After *B. cinerea* cells were exposed to (*E*)-2-hexenal at the MFC for 0, 30, 60, and 120 min, total ergosterol contents were measured by using the previous method with slight alterations (Tao et al., 2014). A measure of 0.0050 g ergosterol standard sample was accurately weighed and dissolved in 95% ethanol. The volume was then constant to final volume of 50 mL, and the concentration of ergosterol liquid storage solution was 0.1 mg mL^–1^. The absorbance value A was determined at 282 nm using a TU-19 UV/VIS spectrophotometer (Purkinje General Instrument Co, Ltd. Beijing, China). According to the absorbance value A obtained, the mass concentration of solution C was determined by the standard curve. Control was the samples without (*E*)-2-hexenal treatment.

### Statistical analysis

All data were represented as mean values in triplicate and + standard deviation (S.D.) and were analyzed using Excel 2007 (Microsoft Co., Redmond, WA, United States) to produce the figures. Analysis of variance (ANOVA), followed by Duncan’s multiple range test, was employed for statistical evaluation. The data were processed using SPSS (version 18.0; IBM, Chicago, IL, United States).

## Results

### Extracellular conductivity

**FIGURE 1:**
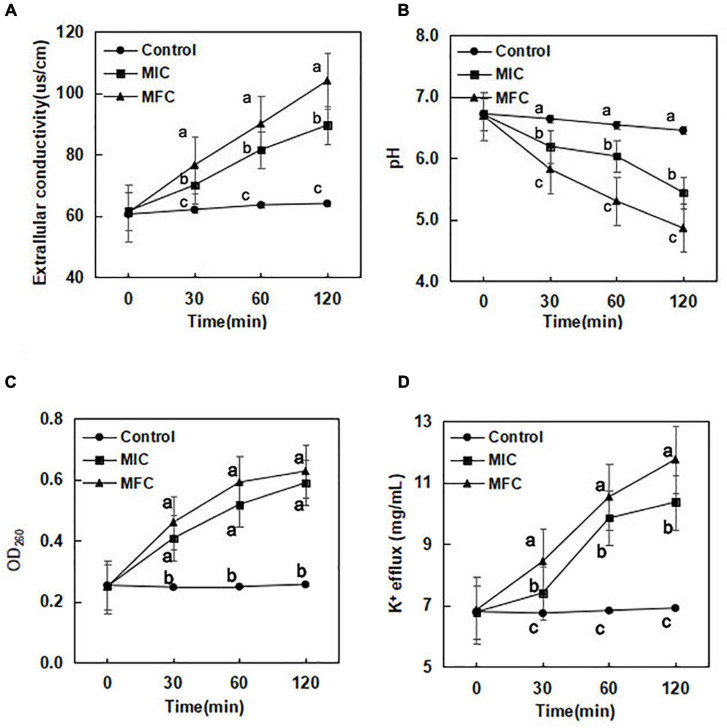
Effect of (*E*)-2-hexenal on extracellular conductivity **(A)**, extracellular pH **(B)**, 260 nm absorbing material release **(C)**, and K^+^ efflux **(D)** of *B. cinerea* [(◆):1 × MIC; (■):1 × MFC; (●): control]. Data presented are the means of pooled data. Error bars indicate the SDs of the means (*n* = 6). Symbols with different letters at each time point indicate significant differences according to Duncan’s multiple range test (*p* < 0.05).

The results of extracellular conductivity from *B. cinerea* cells exposed to (*E*)-2-hexenal (0, 1 × MIC, and 1 × MFC) for a period of 0–120 min are shown in Figure 1A. When exposed to (*E*)-2-hexenal at 1 × MIC and 1 × MFC for 30 min, the values of extracellular conductivity were 70.0 and 76.5 μs/cm, respectively, significantly higher than those of the control (62.0 μs/cm) (*P* < 0.05). The extracellular conductivity increased with the increase in the treatment concentration and exposure time. At 120 min of exposure, the extracellular conductivity of *B. cinerea* suspensions with (*E*)-2-hexenal at 1 × MIC and 1 × MFC obviously increased, with conductivity reaching 89.5 μs/cm for 1 × MIC and 104.0 μs/cm for 1 × MFC, respectively. However, the change in extracellular conductivity for control (64.0 μs/cm) was almost unchanged.

### Extracellular pH

The extracellular pH of *B. cinerea* cells exposed to (*E*)-2-hexenal is presented in Figure 1B. A downward trend in extracellular pH was observed for the control, 1 × MIC, and 1 × MFC. A sharp decline in extracellular pH occurred at 1 × MFC and 1 × MIC. After incubation with (*E*)-2-hexenal at 1 × MIC and 1 × MFC for 30 min, the extracellular pH values in *B. cinerea* suspensions were 6.19 and 5.82, respectively, which were obviously lower than those of control (6.64) (*P* < 0.05). The extracellular pH values in *B. cinerea* suspensions after incubation with (*E*)-2-hexenal at 1 × MIC and 1 × MFC for 120 min were 5.42 and 4.82, respectively, which were significantly lower than those of control (6.45) (*P* < 0.05).

### DNA release

The results of the release of DNA when *B. cinerea* was treated with (*E*)-2-hexenal at three different concentrations (0, 1 × MIC, and 1 × MFC) for 0, 30, 60, and 120 min, respectively, are shown in Figure 1C. After *B. cinerea* cells were treated with (*E*)-2-hexenal at different concentrations, an immediately significant (*P* < 0.05) increase in the release of DNA was observed. The OD_260_ values in *B. cinerea* suspensions without (*E*)-2-hexenal remained constant throughout the treatment period. The OD_260_ value in the *B. cinerea* suspensions treated with (*E*)-2-hexenal at 1 × MIC for 120 min was 0.59, the value was much higher (*P* < 0.05) than that of control (0.26) and slightly lower than that at 1 × MFC (0.63).

### Potassium ion efflux

Potassium ions (K^+^) leaked from *B. cinerea* mycelia when incubated with (*E*)-2-hexenal (Figure 1D). (*E*)-2-Hexenal at 1 × MFC could significantly induce K^+^ release during the initial 30 min of treatment course, and the potassium ion concentration was 8.43 μg/mL after (*E*)-2-hexenal at 1 × MFC was treated for 30 min. When the processing time reached 120 min, K^+^ release at 1 × MFC of (*E*)-2-hexenal continuously increased and reached 11.75 μg/mL. By comparison, treatment with (*E*)-2-hexenal at 1 × MIC (10.36 μg/mL) resulted in more K^+^ release than control (6.91 μg/mL) after *B. cinerea* mycelia was incubated for 120 min.

### Effect of (*E*)-2-hexenal on the mycelium and spores of surface morphology of *Botrytis cinerea*

**FIGURE 2:**
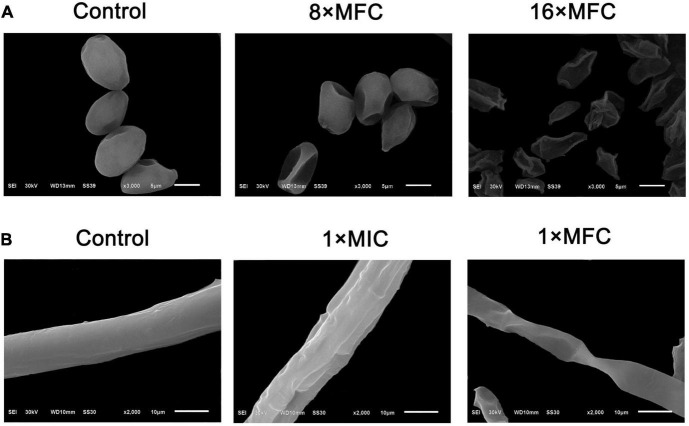
Scanning electron microphotography of *B. cinerea* spores and hyphae after treatment with (*E*)-2-hexenal. **(A)** Spores of untreated, 8 × MFC, and 16 × MFC (*E*)-2-hexenal; **(B)** Mycelia of untreated, 1 × MIC (160 μL/L), and 1 × MFC (320 μL/L) (*E*)-2-hexenal.

Scanning electron microscopy images showed treatment with (*E*)-2-hexenal at 8 × MFC or 16 × MFC for 2 h obviously altered the surface morphology of *B. cinerea* spores (Figure 2A). The use of high concentrations of (*E*)-2-hexenal (8 × MFC and 16 × MFC) was determined by repeated verification using tomato inoculation experiments (data not shown). The effects of (*E*)-2-hexenal on the surface morphology of *B. cinerea* mycelia could be obtained by observing the surface ultrastructure by SEM (Figure 2B). Particularly, compared with control, when treated with (*E*)-2-hexenal at 8 × MFC, each spore displayed depressed and shrunken spore surfaces. By contrast, (*E*)-2-hexenal at 16 × MFC disposed *B. cinerea* spores after 2 h of exposure, which appeared severely collapsed, and most of these spores had ruptured because of the lack of cytoplasm. At the same time, compared with control, *B. cinerea* hyphae subjected to (*E*)-2-hexenal at 1 × MIC for 2 h displayed some changes in surface morphology, showing a little shrinkage; however, it was still smooth and full. However, when the hyphae was treated with 1 × MFC of (*E*)-2-hexenal, the severe morphological changes have occurred, the hyphae had twisted, shrunk, and even ruptured.

### Effect of (*E*)-2-hexenal on the plasma membrane integrity of *Botrytis cinerea* spores

**FIGURE 3:**
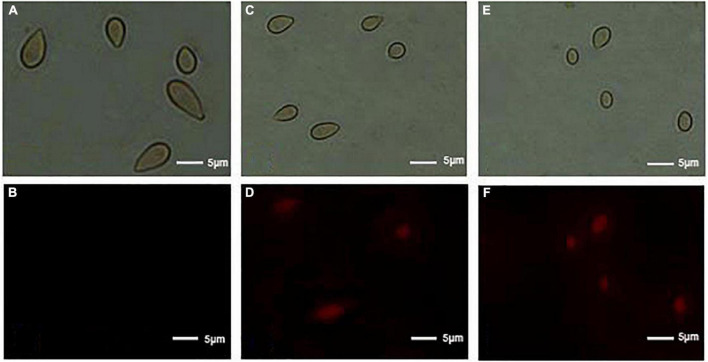
Effect of control, 8 × MFC, and 16 × MFC (E)-2-hexenal on the membrane integrity of *B. cinerea* spores. Spores were cultured at 28 + 2°C in PDB medium supplemented with 0 (**A,** bright field; **B,** PI) or 8 × MFC (**C,** bright field; **D,** PI), and 16 × MFC (**E,** bright field; **F,** PI) for 2 h of incubation, and spores were stained with the PI and observed with a fluorescence microscope. The scale bar indicated the length of 5 μm.

In order to evaluate the action mode of (*E*)-2-hexenal on *B. cinerea* spores, the integrity of *B. cinerea* spore cell membranes was measured using an Axioskop 40 fluorescence Zeiss microscope (Zeiss, Oberkochen, Germany). Their plasma membranes were distinctly destroyed by (*E*)-2-hexenal. These were positively correlated with reagent concentration and processing time (Figure 3). Untreated control emerged to have intact plasma membranes, whereas macroscopic observation by using a fluorescence microscope showed that all the spore plasma membranes with (*E*)-2-hexenal at 16 × MFC were destroyed. However, several spore plasma membranes with (*E*)-2-hexenal at 8 × MFC were damaged. The experimental results of PI staining and SEM showed that the integrity of the *B. cinerea* plasma membrane distinctly decreased with the increase in reagent concentration (Figures 2, 3).

### Overall transcriptome profiles *Botrytis cinerea*

In order to determine their transcriptome profiles of *B. cinerea* in CK and (E)-2-hexenal-treated groups, a congregated cDNA specimen from each group was sequenced separately using the Illumina sequencing platform. The concrete contents in sequence assembly and information annotation are presented in Table 2. After the raw reads were filtered, 23.6 and 23.1 million clean reads were accessible from CK and (*E*)-2-hexenal, respectively. A total of 288,357 unigenes were entirely utilized for functional annotation. Among them, 107,120 unigenes were annotated to the GO database. The total number of identified DEGs were 3,893 between CK and (*E*)-2-hexenal, which included 1,949 upregulated and 1,944 downregulated genes (Table 2). The number of upregulated genes was almost equal to that of downregulated genes.

**TABLE 2:** Summary data of reads in control (CK) and (*E*)-2-hexenal-treated *B. cinerea* mycelia transcriptomes.

Parameters	Control	*Trans*-2-hexenal	Total
Total clean reads	23,601,589	23,125,970	
Total clean nucleotides (nt)	7,080,476,700	6,937,790,850	
Q20 (%)	97.8	97.95	
Q30 (%)	93.38	93.45	
GC (%)	47.7	47.3	
Up-regulated genes			1949
Down- regulated genes			1944
Total different expressed gene			3893
Unigenes annotation against GO			15782
Unigenes annotation against KEGG			2113
Total genes	11940	11883	

**FIGURE 4:**
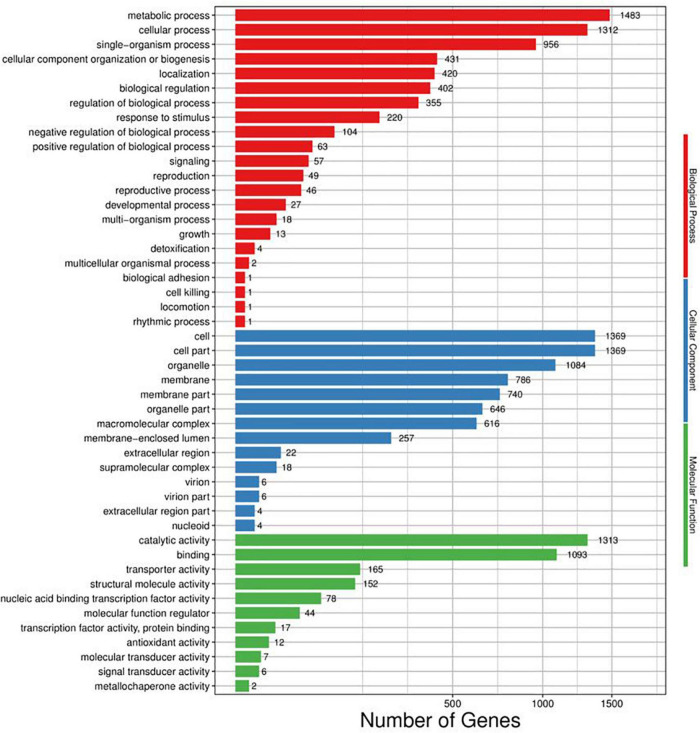
Distribution of genes differentially regulated in response to (*E*)-2-hexenal in the three main categories. The number of DEGs belonging to the most represented categories and the *P*-values of gene ontology enrichment are shown.

Web Gene Ontology Annotation Plot (WEGO) was utilized to visualize and plot the GO annotation. Annotated unigenes were classified by GO assignments into three ontologies with large amounts of cellular component (6,927), followed by biological process (5,366), and molecular function (2,889) (Figure 4). In metabolic process, single-organism metabolic process, phosphate-containing compound metabolic process, and phosphorus metabolic process are the most enriched functional groups with 583, 179, and 188 unigenes, respectively. In molecular function, binding and catalytic activities were the top two groups. Cell part, cell, and organelle in cellular components compose the highest percentage, together with unigenes abundance in the cytoplasm (943) maximally, membrane (786), integral component of membrane (679), nucleus (611), etc.

**FIGURE 5:**
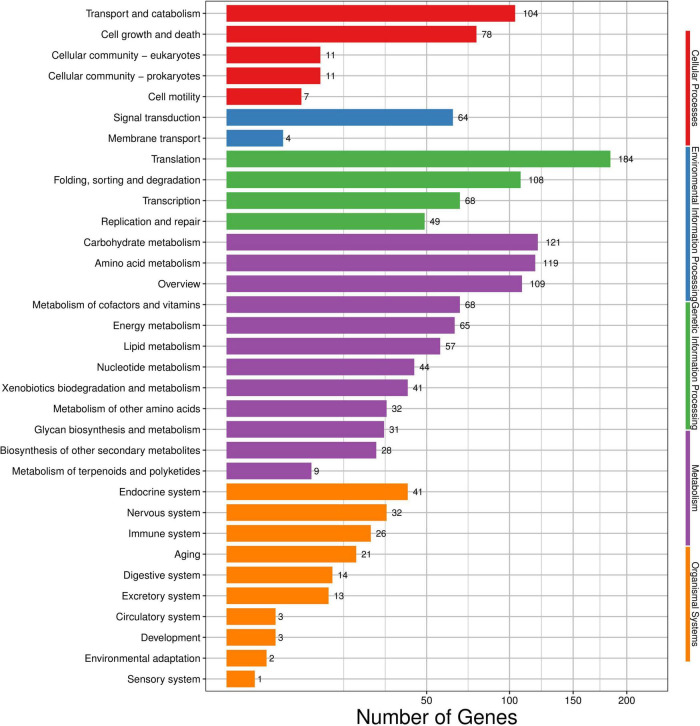
Kyoto encyclopedia of genes and genomes (KEGG) categories mapped from the annotated proteins. The vertical axis lists the names of pathways in the KEGG database, and the horizontal axis shows the proportion of annotated genes in each pathway.

Among these DEGs, 2,113 DEGs in total were mapped to the KEGG database and added up to 259 pathways (Figure 5). The concrete pathways related to environmental information processing, such as ABC transporters (2, 0.09%), plant–pathogen interaction (1, 0.05%), MAPK signaling pathway (29, 1.37%), and phosphatidylinositol signaling (6, 0.28%); some pathways associated with the cell membrane, such as steroid biosynthesis (9, 0.43%), fatty acid biosynthesis (6, 0.28%), and amino acid biosynthesis (62, 2.93%); some pathways related to energy, citrate cycle (4, 0.19%), starch and sucrose metabolism (17, 0.80%), oxidative phosphorylation (23, 1.09%), and glycolysis/gluconeogenesis (17, 0.80%); and some pathways involved in genetic information, such as RNA degradation (22, 1.04%), RNA polymerase (11, 0.52%), ribosome biogenesis in eukaryotes (13, 0.62%), ribosomes (97, 4.59%), and aminoacyl-tRNA biosynthesis (30, 1.42%), were all impacted by the treatment of (*E*)-2-hexenal (Supplementary Table S1).

### Genes associated with cell wall biosynthesis

The cell wall is the outer layer of the cell membrane which protects the cell from osmotic strength and mechanical injures and establishes the cell shape. We discovered that several pathways related to cell wall biosynthesis (Supplementary Table S2), such as nucleotide sugar and amino sugar metabolism, and sucrose and starch metabolism, were prominently affected by (E)-2-hexenal. *Chitin synthase 1* (*CHS1*), *chitin synthase 5* (*CHS5*), and *1,3-beta-glucan synthase 1* (*FKS1*), three crucial cell wall-related genes which encoded for 1,3-β-glucan synthase and chitin synthase, respectively, were predominantly activated. The results obtained in this experiment were in accord with other previous research.

### Genes associated with the cell membrane

Several genes were involved in pathways associated with cell membrane metabolism (Supplementary Table S3), such as fatty acid biosynthesis and metabolism, inositol phosphate metabolism, steroid biosynthesis, and unsaturated fatty acid biosynthesis, and their expression levels were altered after exposed to (*E*)-2-hexenal. Among them, *inositol monophosphate 1-phosphatase* (*IMPA*), *phosphatidylinositol 3-kinase VPS34* (*PIK3C3*), *acetyl-CoA acetyltransferase 1* (*ACAA1)*, *enoyl-[acyl-carrier-protein] reductase 1* (*NRBF1*), *lanosterol synthase* (*ERG7*), and 3-keto-steroid reductase (*ERG27*) were downregulated, indicating that the integrity of the cell membrane might be destructed. In the current study, (*E*)-2-hexenal severely disrupted ergosterol biosynthesis in *B. cinerea* cells (Supplementary Table S4). These results demonstrate that these plasma membranes were a crucial antifungal target for (*E*)-2-hexenal. Remarkably, the expression quantities of four genes in the biosynthetic pathway of ergosterol, which encoded for *lanosterol synthase* (*ERG7*), *mevalonate kinase* (*ERG12*), *hydroxymethylglutaryl-CoA synthase* (*ERG13*), and *3-keto-steroid reductase* (*ERG27*), were downregulated 6. 3-, 5. 0-, 3. 7-, and 2.9-fold, respectively. On the contrary, expression quantity of *3-ketoacyl-(acyl-carrier-protein) reductase* (*fabG*) was enhanced 2.91-fold.

### Effect of (*E*)-2-hexenal on gene expression levels in ergosterol biosynthesis

**FIGURE 6:**
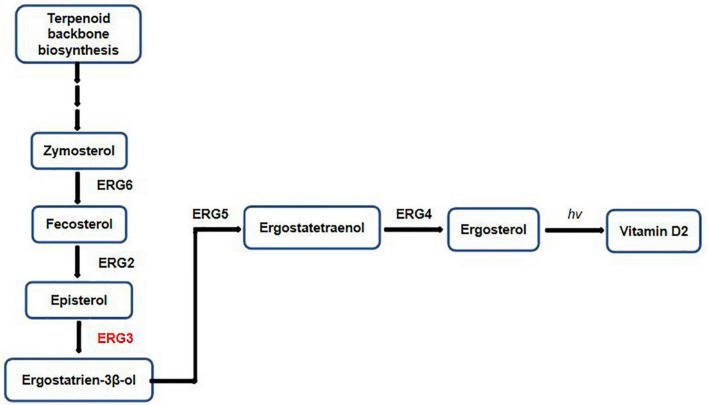
Factors and intermediates of the fungal ergosterol biosynthetic pathway.

In order to determine the reliability of transcriptome expression levels, the ergosterol-related biosynthetic genes were selected for qRT-PCR analysis. These candidate genes play indispensable roles in the biosynthetic pathway of ergosterol (Figure 6). The terpenoid backbone underwent a series of enzymatic reactions by regulating distinct downstream targets to form ergosterol under the catalysis of *ERG* gene families.

**FIGURE 7:**
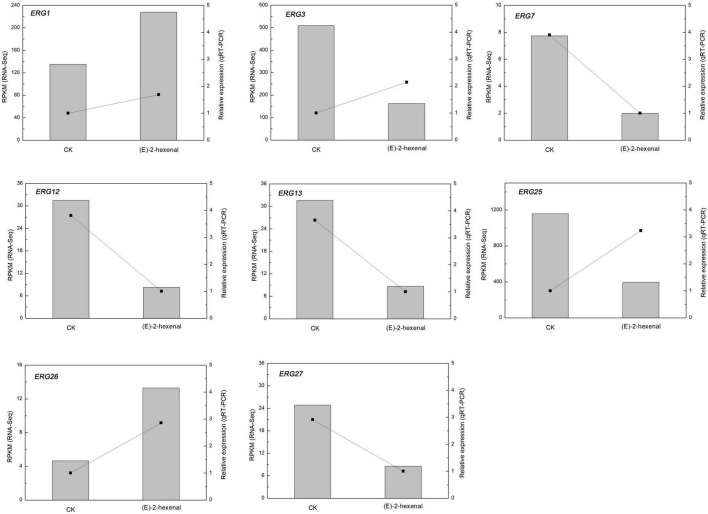
Changes in the expression of ergosterol biosynthesis genes of *B. cinerea* mycelia treated by CK and 1 × MFC (*E*)-2-hexenal for 2 h. Data from qRT-PCR were normalized relative to the geometric average of *BcRPL5*. The relative expression level of each gene in CK was set at 1, and the values are the mean + SD of three replications. The normalized expression level (RPKM) of RNA-Seq is indicated on the *y*-axis.

As shown in Figure 7, the candidate genes were either activated or inhibited; however, their expression levels were different in control and the treated samples with (*E*)-2-hexenal. *ERG7* and *ERG27* gene expressions in control and the processed samples were repressed, with a higher expression level found in control than that in the processed samples. By contrast, *delta(7)-sterol 5(6)-desaturase* (*ERG3*) and *methylsterol monooxygenase* (*ERG25*) gene expressions were markedly induced by (*E*)-2-hexenal, and their expression levels were dramatically increased compared to those in control groups, with 3.14- and 2.93-fold, respectively (Figure 7). Most results were in concordance with RNA-Seq data, which manifesting that RNA-Seq analysis was valid, although the fold change of several detected unigenes by qRT-PCR and sequencing did not exactly match. The expression levels of *ERG3* and *ERG25* by qRT-PCR analysis were upregulated, whereas the levels were inhibited in RNA-Seq data. Taken together, it can be inferred that (*E*)-2-hexenal is able to impact the expression quantities of ergosterol biosynthetic gene pathways in *B. cinerea* cells.

### Effect of (*E*)-2-hexenal on ergosterol contents

**FIGURE 8:**
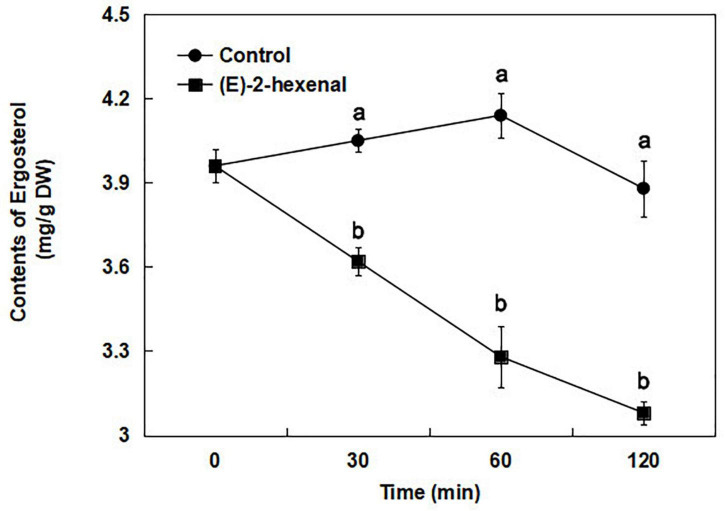
Total ergosterol contents in *B. cinerea* control (●) and 1 × MFC (*E*)-2-hexenal (■) treated cells, and values are the mean + SD of three measurements. Symbols with different letters at each time point indicate significant differences according to Duncan’s multiple range test (*p* < 0.05).

To verify RNA-Seq analysis and to account for the influence of (E)-2-hexenal on ergosterol biosynthesis further, the ergosterol contents of *B. cinerea* were determined by using a UV/VIS spectrophotometer. According to the ergosterol standard curve, the ergosterol content in the control and 1 × MFC (*E*)-2-hexenal-treated samples was determined by measuring the absorbance value at 282 nm. The ergosterol contents of *B. cinerea* cells treated with (*E*)-2-hexenal continuously decreased throughout the period, whereas those in the untreated cells were almost constant (Figure 8). The ergosterol content of *B. cinerea* cells exposed to 1 × MFC of (*E*)-2-hexenal for 30 min was 3.62 + 0.05 mg/g DW, which was significantly lower than that of control (4.05 + 0.04 mg/g DW). The difference increased with the extension of treatment time. When the incubation time was 120 min, the ergosterol content in treatment cells was 3.08 + 0.04 mg/g DW, as compared to 3.88 + 0.10 mg/g DW in control cells.

### Gene expression associated with energy metabolism

In our study, most of pyruvate-related metabolic genes were downregulated after treatment with (*E*)-2-hexenal, implying that downstream pathways of pyruvate might be inhibited (Supplementary Table S5), such as *pyruvate carboxylase* (*pyc*), *D-lactate dehydrogenase* (*LDHD*), *pyruvate kinase* (*pyk*), *alpha-isopropylmalate synthase* (*leuA*), *dihydrolipoamide succinyltransferase* (*pdhC*), and *glyoxalase* (*gloA*). However, during the pyruvate metabolic process, individual genes are upregulated, such as *malate dehydrogenase* (*maeB*), *phosphoenolpyruvate carboxykinase* (*pckA*), and *alpha-isopropylmalate synthase* (*LYS20*).

Almost all the acetyl-CoA related to genes were decreased by (*E*)-2-hexenal, indicating that energy metabolism of *B. cinerea* in the mitochondria has been interrupted including *acetyl-CoA acetyltransferase 1* (*ACAA1*), *pyruvate dehydrogenase* (*pdhC*), *pyruvate carboxylase* (*pyc*), *biotin holocarboxylase synthetase* (*HLCS*), and *N-acetyl-gamma-glutamyl-phosphate reductase* (*ARG56*). In this research, (*E*)-2-hexenal might also interrupt oxidative phosphorylation and the tricarboxylic acid cycle (TAC) pathway in *B. cinerea*, leading to the decrease in protein biosynthesis and the ATP content, which are indispensable for hyphal normal growth.

Furthermore, mitochondrial dehydrogenases, which comprise a variety of crucial dehydrogenases for ATP formation such as *pyruvate dehydrogenase* (*pdhC*), *malate dehydrogenase* (*RPL28*), *aldehyde dehydrogenase* (*gabD*), and *succinate dehydrogenase* (*DDC*), were immensely suppressed by (*E*)-2-hexenal. This also affords strong evidence of mitochondrial dysfunction.

## Discussion

It was previously reported that (*E*)-2-hexenal, as a surfactant, is likely permeated by passive diffusion across the plasma membrane. Once (*E*)-2-hexenal enters cells, biologically crucial nucleophilic groups react with its α, β-unsaturated aldehyde moiety (Patrignani et al., 2015). However, the antimicrobial mechanisms of some other aldehydes, such as ortho-phthalaldehyde and glutaraldehyde, are likely to enhance its permeability and participate in interaction with the cytoplasmic membrane (Zi et al., 2018). Previous studies have shown that (*E*)-2-hexenal has antifungal activity with MIC 160 μL/L and MFC 320 μL/L, which may be relevant to the fungal membrane damage (Zhang et al., 2017).

Using the RNA-Seq method, Reeder et al. (2017) compared gene expression of *Pseudogymnoascus destructans* grown in the culture medium, and they were surprised to find that gene expression of the subtilisin protease family was significantly reduced during infection. Korn et al. (2021) suggested that *trans*-2-hexenal was a safe and known volatile compound that inhibit the growth of plant pathogens, and its presence led to the downregulation of many genes that were thought to be involved in the virulence of *P. destructans*.

Ergosterol is the key component of fungal lipid membranes and is responsible for maintaining the membrane fluidity and cell integrity (Tian et al., 2015). Commercial fungicides interrupt normal biosynthesis of sterol substances or markedly decrease the amount of ergosterol in *Penicillium italicum* (Ruan et al., 2017). Ergosterol has also the responsibility to sustain cell integrity and function (Tian et al., 2012; Xu et al., 2017). Fungi alleviate the damage cause by the ergosterol inhibitor to the cell membrane by upregulating the expression of ergosterol biosynthesis-related genes (Yang et al., 2020). *ERG11* in *A. fumigatus*, which was one of genes associated with the ergosterol biosynthesis pathway, was upregulated under amphotericin B pressure (Gautam et al., 2008).

In the current study, several genes might be important candidate genes involved in ergosterol biosynthesis genes, such as *ERG1*, *ERG3*, *ERG7*, *ERG12*, *ERG13*, *ERG25*, *ERG26*, and *ERG2*7, and most of them were suppressed (Figure 7). The qRT-PCR results of *ERG3* and *ERG25* were inconsistent with RNA-Seq sequencing results. The probable reasons were in connection with a number of factors, for instance, selection of reference genes and error in manual operation. These experimental results of the expression quantities in the biosynthetic pathway of ergosterol were associated with previous findings.

The decrease in energy metabolism may be a response to or a feedback adverse environmental condition to maintain cell viability (Wang et al., 2018). Natural phytochemicals not only destabilized the cell membranes but also impeded the energy transformation process of the fungus, causing ATP to leak from the fungal mycelium (Leyva Salas et al., 2017). To investigate the in-depth mechanism of possible antifungal actions of (*E*)-2-hexenal, RNA sequencing was performed to determine its effect on *B. cinerea*.

Gene ontology term annotation and KEGG pathway enrichment analysis showed that a number of significantly enriched DEGs may be related to the stimulation of concomitant mitochondrial dysfunction and early apoptosis, such as disruption of pyruvate metabolism and reduced ATP synthesis and acetyl-CoA and dehydrogenase activity (Supplementary Tables S3–S5). These results depicted the process of cell membrane damage was enslaved to energy deficit of *B. cinerea* caused by (*E*)-2-hexenal treatment, which is thought of as the possible antifungal mechanism of (*E*)-2-hexenal against *B. cinerea* by distraction of the TAC of hyphal cells, leading to energy deficiency and cell membrane damage, ultimately accelerating cell death.

In order to survive from extreme environmental changes, eucaryons must swiftly modulate their gene expression patterns to produce a battery of defense mechanisms (Legay et al., 2011; Li et al., 2011). In this research, (*E*)-2-hexenal caused significant changes in gene expression amounts associated with defense, detoxification, and cell rescue. At the same time, (E)-2-hexenal is proved to effectively inhibit the mycelial growth of pathogenic fungi in a dose-dependent manner. Genes related to the over-represented pathway, such as mRNA surveillance pathway, ribosome biogenesis in eukaryotes, RNA transport and aminoacyl-tRNA biosynthesis, and RNA polymerase, were incompletely depressed, manifesting a harm in these cellular translational activities (Supplementary Table S6). Furthermore, one gene liable for mitochondrial ribosomal protein (*MRPS5*) was found to be downregulated, indicating a block in mitochondrial dysfunction and mitochondrial translation (Leeuwen et al., 2013).

In the same way, an interruption in energy-associated pathways including TCA, oxidative phosphorylation, gluconeogenesis/glycolysis, nitrogen metabolism, and sulfur metabolism was also discovered. Most of the genes in the aforementioned pathways, such as *pyruvate carboxylase* (*PYC*), *isocitrate dehydrogenase 2* (*IDH2*), *dihydrolipoamide succinyltransferase* (*DLAT*), enolase (*ENO*), *3-phosphoglycerate kinase* (*PGK*), *2,3-bisphosphoglycerate-independent phosphoglycerate mutase I* (*gpmI*), *ATP synthase 4* (*ATP4*), *NADH dehydrogenase (ubiquinone) 8* (*NDUFB8*), *NADH:ubiquinone oxidoreductase 2* (*NDUFV2*), *cysteine synthase 1* (*cysK*), *cystathionine gamma-synthase* (*metB*), and *glutamate-ammonia ligase* (*glnA*), were downregulated (Supplementary Table S6).

Previous studies have demonstrated that (*E*)-2-hexenal could visibly transform the mitochondrial morphology, restrain the TCA cycle, and reduce 15.93% ATP content of *Penicillium digitatum* at MFC for 120 min (Zheng et al., 2015). Under extreme environmental conditions, the downregulated energy-requiring processes are a very crucial method to get rid of energy crisis and keep the cells alive (Stasolla, 2010).

The cell membranes maintain functions and cell shapes and are one of the primary targets of essential oils (EOs) to describe their antifungal potential (Siroli et al., 2015). These structural changes in several targets including cell wall, mitochondrion, cell membrane, and inherited matter, have been put forward to illuminate the antifungal activity of essential oils or their volatile ingredients (Shao et al., 2013; Fabra et al., 2016). Salem et al. (2016) reported that these genes were sharply induced when *S. cerevisiae* cells were exposed to α-terpinene and stimulated compensatory mechanism of cytoderm to conquer the toxicity of terpinene. Terpenoid has the lipophilic property, so it can preferentially enter into the lipid membrane, which leads to increasing fluidity of the bilayer lipid membrane and ultimately increasing membrane permeability (Xu et al., 2017). (*E*)-2-hexenal could constrain fungal pathogens by disrupting the integrity and permeability of the cell membrane (Ma et al., 2019).

These data provide extensive gene expression information and will certainly accelerate the study of ergosterol biosynthesis and mitochondrial dysfunction in *B. cinerea* at the level of transcriptional expression when exposed to (*E*)-2-hexenal.

## Data availability statement

The datasets presented in this study can be found in online repositories. The names of the repository/repositories and accession number(s) can be found in the article/Supplementary material.

## Author contributions

GS and SD conducted the experiments. QL analyzed the data. HW and MY contributed to the manuscript writing and review. JZ designed the experiments and drafted the manuscript. All authors read and approved the manuscript.
